# Comparison of neurosurgical and medical management options of space-occupying cerebellar infarction

**DOI:** 10.1007/s00701-026-06809-3

**Published:** 2026-03-06

**Authors:** Cristian D. Mendieta-Barrera, Pavell Dhondt, Anuraag Punukollu, Fabricio Garcia-Torrico, Diana Laura Ochoa-Hernández, Rômulo da Silva Sanglard, Flor Belén Villalobos-Villalobos, Kevin Mamani-Julian, Luciana Rivera-Hurtado, Roel Meeus, Leonardo Rangel-Castilla, Arash Ghaffari-Rafi

**Affiliations:** 1 Universidad Mayor Real y Pontificia de San Francisco Xavier, Chuquisaca, Bolivia; 2 University Hospitals Leuven, Leuven, Belgium; 3 Andhra Medical College, Visakhapatnam, India; 4 Universidad Mayor de San Andrés, La Paz, Bolivia; 5 Universidad Nacional Autónoma de Mexico, Mexico City, Mexico; 6 Universidade Federal Do Rio de Janeiro, Rio de Janeiro, Brazil; 7 Universidad Autónoma de Coahuila Uadec Unidad Torreón, Mexico City, Mexico; 8 Hospital Lomas International, San Luis Potosí, San Luis Potosí, México; 9 School of Medicine, Department of Neurological Surgery, University of California, Davis, Sacramento, CA USA; 10 Department of Neurological Surgery, School of Medicine, University of California, Davis, 4860 Y Street Suite 3740, Sacramento, CA 95817 USA

**Keywords:** Cerebellar infarction, Suboccipital craniectomy, External ventricular drain, Necrosectomy, Stroke, Medical management

## Abstract

**Background/objectives:**

Despite space-occupying cerebellar infarctions (SOCIs) carrying a high morbidity and mortality due to mass effect in the posterior fossa, optimal management remains uncertain: particularly regarding patient selection, timing, and surgical technique. We conducted a systematic review and network meta-analysis to compare outcomes between medical versus surgical management, and to identify prognostic thresholds that may guide treatment.

**Methods:**

A search of PubMed, Embase, and CENTRAL was performed from database inception through October 30, 2024. Studies were included if they reported outcomes in ≥ 10 patients with SOCI treated with medical management and/or surgical interventions, including suboccipital decompressive craniectomy (SDC), external ventricular drainage (EVD), and necrosectomy. Data extraction and risk-of-bias assessment were performed independently by multiple reviewers. Random-effects meta-analyses and frequentist network meta-analyses were conducted. Primary outcomes were favorable functional status and mortality; secondary outcomes included length of hospital stay.

**Results:**

Eighteen studies comprising 754 patients met inclusion criteria. Surgical intervention was associated with superior outcomes in patients with infarct volumes > 51 mL (61.5% favorable outcome vs. 35.0% with medical therapy; *p* = 0.018) or GCS ≤ 13 (*p* < 0.05). Among surgical strategies, SDC combined with necrosectomy and an EVD (SDC–N–EVD) conferred the greatest probability of favorable outcome (OR 3.1, 95% CI: 1.18–8.14; *p* < 0.05), reduced mortality risk, and shortened length of hospitalization. Neither patient age nor surgical timing within 72 h significantly altered outcomes.

**Conclusions:**

Surgical management, particularly SDC–N–EVD, was associated with improved outcomes compared to medical therapy alone for SOCI, especially in patients with large infarcts or impaired consciousness. Infarct volume and pre-interventional GCS can provide prognostic thresholds. While the inherent heterogeneity of the data indicates these results should be interpreted with caution, they provide impetus for conducting standardized multicenter prospective studies to validate these observations and establish evidence-based treatment algorithms.

**Supplementary Information:**

The online version contains supplementary material available at 10.1007/s00701-026-06809-3.

## Introduction

Space-occupying cerebellar infarctions (SOCIs) represent a small proportion of ischemic strokes (1.5–4.2%), yet are associated with disproportionately high morbidity and mortality rates (15–32%), due to the confined anatomy of the posterior fossa [2, 3, 6, 11, 16, 18, 32]. Despite the high risk of life-threatening complications (i.e., obstructive hydrocephalus, brainstem compression, herniation) and death, there remains no consensus on optimal surgical management strategies for SOCIs [11, 16, 18, 24, 29, 32, 38].

Upon diagnosis, initial medical therapy is focused on mitigating cerebral edema and lowering intracranial pressure [8, 24, 35]. However, in the presence of neurological decline or radiographic progression, surgical intervention becomes necessary, including: external ventricular drainage (EVD), suboccipital decompressive craniectomy (SDC), and necrosectomy [1, 8, 14, 15, 24]. Despite widespread use of these surgical interventions, uncertainties remain, regarding their optimal timing, indications, and long-term effectiveness, with no universally accepted treatment algorithm [24, 29]. Crucially, the existing literature often fails to distinguish between specific surgical nuances—such as the added value of necrosectomy or EVD combined with decompression—, leaving a significant gap in knowledge regarding the comparative efficacy of distinct surgical subtypes [1, 8, 14, 15, 24]. Furthermore, while there is literature to suggest that key clinical (pre-interventional Glasgow Coma Scale [GCS] score) and radiographic (infarct volume, presence of hydrocephalus) parameters may guide therapeutic decisions, these findings are tempered by methodological variability, limited sample sizes, and a paucity of randomized controlled trials, which ultimately hinders the development of standardized care pathways [1, 9, 14, 15, 19, 35].

To address these knowledge gaps, we conducted a systematic review and network meta-analysis comparing the efficacy of medical therapy with surgical interventions for SOCI. Unlike traditional pairwise meta-analyses conducted in the literature, our network approach allows for the simultaneous comparison and ranking of multiple treatment strategies [1, 8, 14, 15, 24]. By synthesizing data from diverse clinical settings and intervention strategies, this study aims to clarify treatment outcomes, identify key prognostic indicators, and inform evidence-based recommendations to support individualized, outcome-driven decision-making in the management of SOCI.

## Methods

This systematic review and network meta-analysis was conducted in accordance with the *Cochrane Handbook for Systematic Reviews of Interventions* and followed the *Preferred Reporting Items for Systematic Reviews and Meta-Analyses* guidelines [5, 20, 26].

### Literature search

A literature search was performed in MEDLINE (PubMed), Embase, and the Cochrane Central Register for Controlled Trials (CENTRAL) from database inception through October 30, 2024. The search strategy combined keywords and MeSH terms relevant to SOCI and associated interventions, including *cerebellar infarction*, *craniectomy*, *craniotomy*, *suboccipital decompressive*, and *necrosectomy*. The full search strategy is provided in Supplementary Methods S1.

### Eligibility criteria

Studies were eligible for inclusion if they met the following criteria: involved patients with SOCI; evaluated surgical interventions (e.g., SOC, EVD, or necrosectomy) with or without medical therapy and/or compared different surgical strategies; reported outcomes related to functional status, mortality, or length of stay; included at least 10 patients. Studies were excluded if they were case reports, abstracts, animal studies, reviews, meta-analyses, or not published in English.

### Data extraction

Data extraction was performed independently by three reviewers (C.D.M.B., A.P., and P.D.) using a standardized form. Extracted variables included study design, country of origin, patient demographics, pre-interventional GCS scores, comorbidities, infarct volume, time from symptom onset to surgery, type of intervention, follow-up duration, and rate of favorable outcomes. Functional outcomes were recorded as defined in the original studies (e.g., Modified Rankin Scale, Glasgow Outcome Scale, etc.).

### Risk of bias assessment

The quality of randomized controlled trials was assessed using the Cochrane Risk of Bias Tool 2.0, while non-randomized studies were evaluated using the Risk Of Bias In Non-randomized Studies of Interventions tool [27, 28]. Highest risk rating across any domain determined the overall study risk. Two reviewers (F.G.T. and C.D.M.B.) independently evaluated each study, with discrepancies resolved through discussion and consensus. Publication bias was evaluated via funnel plots.

### Statistical analysis

#### Linear regression

Associations between favorable outcome rates and pre-interventional variables were explored using linear regression analyses. Independent variables included infarct volume (mL), patient age, pre-interventional GCS score, and time between symptom onset and surgery as independent variables; the dependent variable was favorable outcome rate. The strength of association was assessed using the coefficient of determination (R^2^), and statistical significance was determined using two-tailed p-values. Two-sample z-tests for proportions were applied to evaluate categorical variables.

#### Meta-analysis

For binary outcomes (favorable outcome and mortality), odds ratios (ORs) with 95% confidence intervals (CIs) were calculated using DerSimonian and Laird random-effects models. Heterogeneity was evaluated using the Cochrane Q test and the I^2^ statistic, with an I^2^ > 40% and a *p* < 0.10 defining significant heterogeneity. All meta-analyses were performed using R Studio (version 4.2.3; R Foundation for Statistical Computing, Vienna, Austria) and Review Manager software (RevMan version 5.4.1; The Cochrane Collaboration, London, United Kingdom).

A frequentist random-effects framework was employed to compare multiple treatment strategies simultaneously. Treatments were ranked based on their estimated probability of being the most effective, using the surface under the cumulative ranking curve [25]. Results were presented as league tables, forest plots, and network diagrams.

## Results

### Study selection

**Fig. 1:**
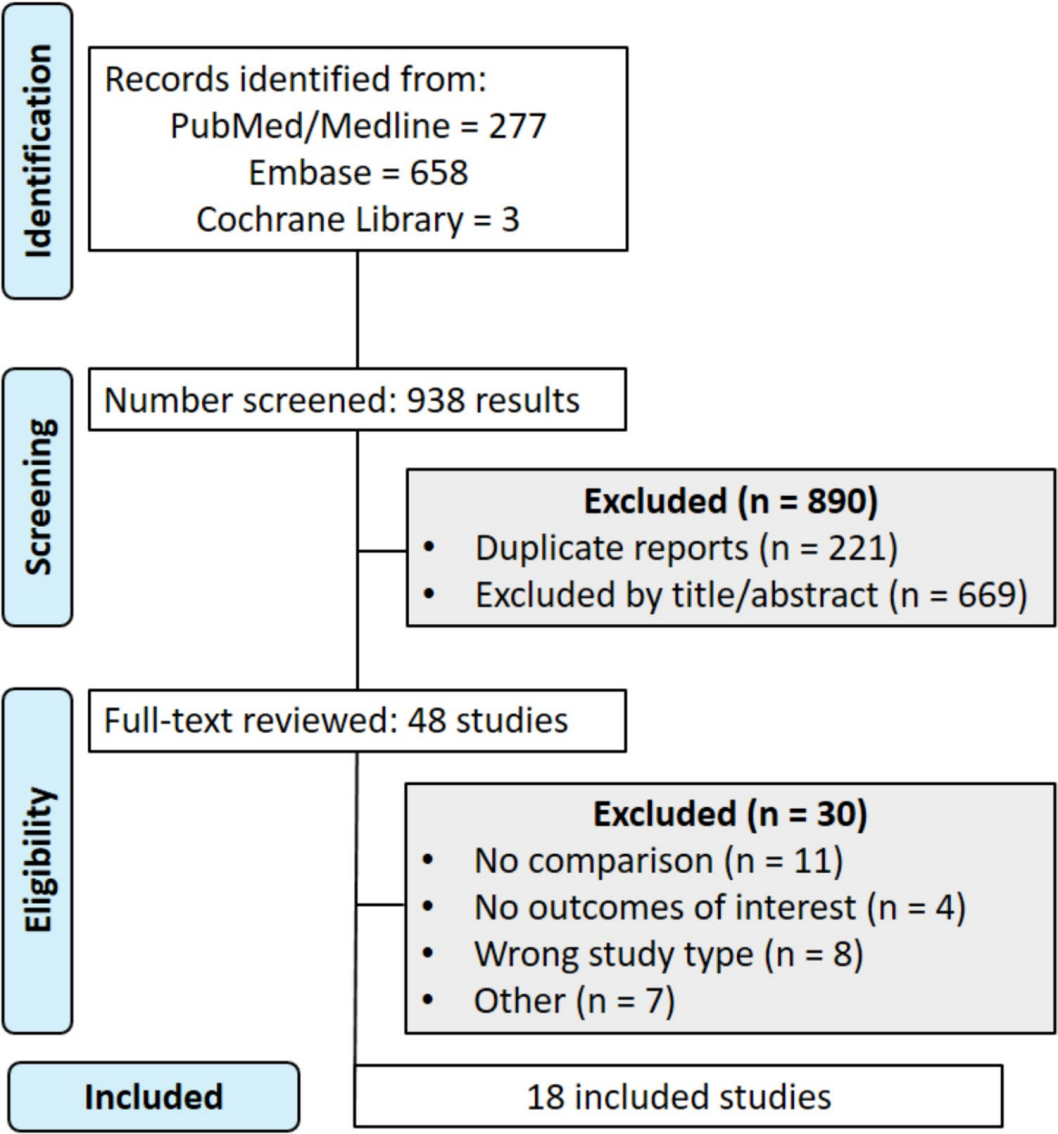
Preferred reporting items for systematic reviews and meta-analyses (PRIMSA) flow diagram. The diagram illustrates the study selection process according to the PRISMA guidelines. A total of 938 records were identified across three databases (PubMed, Embase, and Cochrane Library). After removal of duplicates and title/abstract screening, 48 articles were assessed fully for eligibility. Of these, 30 were excluded based on predefined inclusion and exclusion criteria, resulting in 18 studies included in the final qualitative synthesis

A total of 938 articles were retrieved from database searches (PubMed: 277; Embase: 658; Cochrane Library: 3). After duplicate removal and abstract screening, 890 articles were excluded, leaving 48 for full-text review. Ultimately, 18 studies met the inclusion criteria (Fig. 1).

### Baseline characteristics of cerebellar infarction population

The analysis involved 18 studies published between 1982 and 2024, collectively including 754 patients with SOCI (Germany [*N* = 4 studies], the United States [*N* = 4], Japan [*N* = 4], Italy [*N* = 2], France [*N* = 1], United Kingdom [*N* = 1], Türkiye [*N* = 1], and Germany-Austria [*N* = 1]) [4, 10, 15, 21, 29–31, 36, 39]. Sixteen studies were retrospective, one was prospective study, and one was a randomized controlled trial.

The mean patient age was comparable across groups (67.3 years in the medical cohort vs. 67.1 years in the surgical). Reported mean cerebellar infarct volume was smaller volume in the medical group (20.7 mL) than the surgical group (45.7 mL). Pre-interventional GCS scores were higher among medically managed patients (mean: 13.3) than in the surgical cohort (11). Among patients undergoing surgery, the mean time from symptom onset to surgery was 46.9 h Tables 1 and 2.

**Table 1:** Baseline characteristics of included studies. Characteristics include the study type, national origin, intervention type, follow-up time, and outcome measure utilized. EVD: external ventricular drain; SDC: suboccipital decompressive craniectomy; N: necrosectomy; GCS: Glasgow come score; GOS: Glasgow outcome scale; mRS: Modified Rankin Scale; NR: not reported; RCT: randomized clinical trial

Study	Design	Country	Intervention (n)						Mean Age (Years)		Follow-Up (Months)	Outcome
			Medical	EVD	SDC	EVD + SDC	EVD + SDC + N	SDC + N	Surgical	Medical		
Won, 2024 [39]	Retrospective	Germany	NR	71	NR	NR	NR	71	66.3	NR	12	mRS
Kapapa, 2024 [10]	Retrospective	Germany	21	NR	NR	17	NR	NR	69.5	80.1	NR	mRS
Hernandez-Duran, 2024 [4]	Retrospective	Germany	NR	NR	43	NR	NR	49	65		3	mRS
Kumral, 2023	Prospective (RCT)	Türkiye	40	NR	NR	NR	32	NR	64.7	66.6	12	mRS
Wang, 2022 [36]	Retrospective	United States	112	38					60	60	NR	Discharge Destination
Taylor, 2020 [31]	Retrospective	United States	65	2	NR	9	NR	12	58.5		NR	NR
Suyama, 2018	Retrospective	Japan	NR	NR	5	9	NR	NR	65		3	mRS
Tartara, 2018 [30]	Retrospective	Italy	NR	NR	2	9	NR	NR	64.7		33.8	mRS
Mostofi, 2013 [21]	Retrospective	France	28	6	16	3	NR	NR	59.7	62.4	NR	GCS
Juttler, 2009 [9]	Retrospective	United States	NR	9	NR	39	NR	8	60		36	mRS
Kudo, 2007 [14]	Retrospective	Japan	NR	3	2	3	14	3	62.6		NR	GOS
Raco, 2003 [24]	Retrospective	Italy	25	8	4	5	NR	NR	56		NR	GOS
Koh, 2000	Retrospective	USA	26	6	2	1	NR	NR	NR		16	mRS
Jauss, 1999 [8]	Prospective	Germany-Austria	36	14	30	4	NR	NR	58.5		3	mRS
Mathew, 1995 [19]	Retrospective	United Kingdom	34	7	2	NR	NR	NR	57		NR	GOS
Hornig, 1994 [6]	Retrospective	Germany	16	NR	NR	NR	NR	30	61.2		NR	mRS
Auer, 1986	Retrospective	Japan	32	1	NR	7	NR	NR	61		NR	NR
Taneda, 1982	Retrospective	Japan	5	NR	NR	3	NR	7	59		NR	Complete Recovery

**Table 2:** Baseline characteristics of included studies. Variables included the following: medical comorbidities, cerebellar infarct volume, time to surgery from symptom onset, and pre-interventional GCS. For cells with values separated by a slash, the total patient population was subcategorized by those who received medical therapy versus surgical therapy (the first value represents patient who received medical therapy, the second value for surgical therapy) AF: atrial fibrillation; HTN: arterial hypertension; DM: diabetes mellitus; GCS: Glasgow coma score; NR: not reported

Study	Patients (n)		Medical Comorbidities (n)				Hydrocephalus (n)	Infarct Volume (mL)		Time to Surgery (Hours)	Pre-Intervention GCS	Medical Therapy Utilized
	Medical	Surgical	HTN	DM	Dyslipidemia	AF		Medical	Surgical			
Won, 2024 [39]	NR	142	NR/115	NR/42	NR	NR/32	NR	NR	39.9	26.4	NR/10.3	NR
Kapapa, 2024 [10]	21	17	17/16	3/7	NR	NR	NR	25.8	52.8	48.53	13.3/10.5	Oxygenation, Prophylactic Anticoagulation
Hernandez-Duran, 2024 [4]	NR	92	NR/80	NR/28	NR	NR/24	NR	NR	45.5	27	NR/10.5	NR
Kumral, 2023	40	32	24/21	17/12	13/11	16/12	NR	96.4	98.4	NR	12.8/8.4	Antihypertensives, Anti-Edema Agents, Prophylactic Anticoagulation
Wang, 2022 [36]	112	38	88/28	48/17	43/18	15/8	NR	11.4	25.9	62.4	14/13.5	Anti-Edema Agents
Taylor, 2020 [31]	65	21	48/15	26/11	25/10	NR	5/11	25	46	NR	13.3/13	Anti-Edema Agents
Suyama, 2018	NR	14	NR/9	NR/4	NR	NR/8	NR/12	NR	64.3	NR	NR	NR
Tartara, 2018 [30]	NR	11	NR	NR	NR	NR	NR	NR	NR	36.8	NR/13.6	NR
Mostofi, 2013 [21]	22	16	NR	NR	NR	NR	NR	NR	NR	NR	12.9/11.2	Antihypertensives, Anti-Edema Agents, Antiemetics
Juttler, 2009 [9]	NR	56	NR	NR	NR	NR	NR	NR	NR	72	NR/13	NR
Kudo, 2007 [14]	NR	25	NR	NR	NR	NR	NR	NR	NR	55.2	NR/6	NR
Raco, 2003 [24]	25	17	NR	NR	NR	NR	NR	NR	NR	NR	NR	Anti-Edema Agents, Prophylactic Anticoagulation
Koh, 2000	26	9	NR	NR	NR	NR	NR/9	NR	NR	NR	NR	Anti-Edema Agents, Hyperventilation
Jauss, 1999 [8]	36	48	NR	NR	NR	NR	NR	NR	NR	NR	NR	Anti-Edema Agents
Mathew, 1995 [19]	34	9	NR	NR	NR	NR	NR	NR	NR	NR	11	Anti-Edema Agents
Hornig, 1994 [6]	16	30	NR	NR	NR	NR	NR	NR	NR	NR	NR	Anti-Edema Agents
Auer, 1986	32	8	NR	NR	NR	NR	NR	NR	NR	NR	NR	NR
Taneda, 1982	5	10	NR	NR	NR	NR	15	NR	NR	NR	NR	NR

### Variables associated with outcome

#### Cerebellar infarct volume

**Fig. 2:**
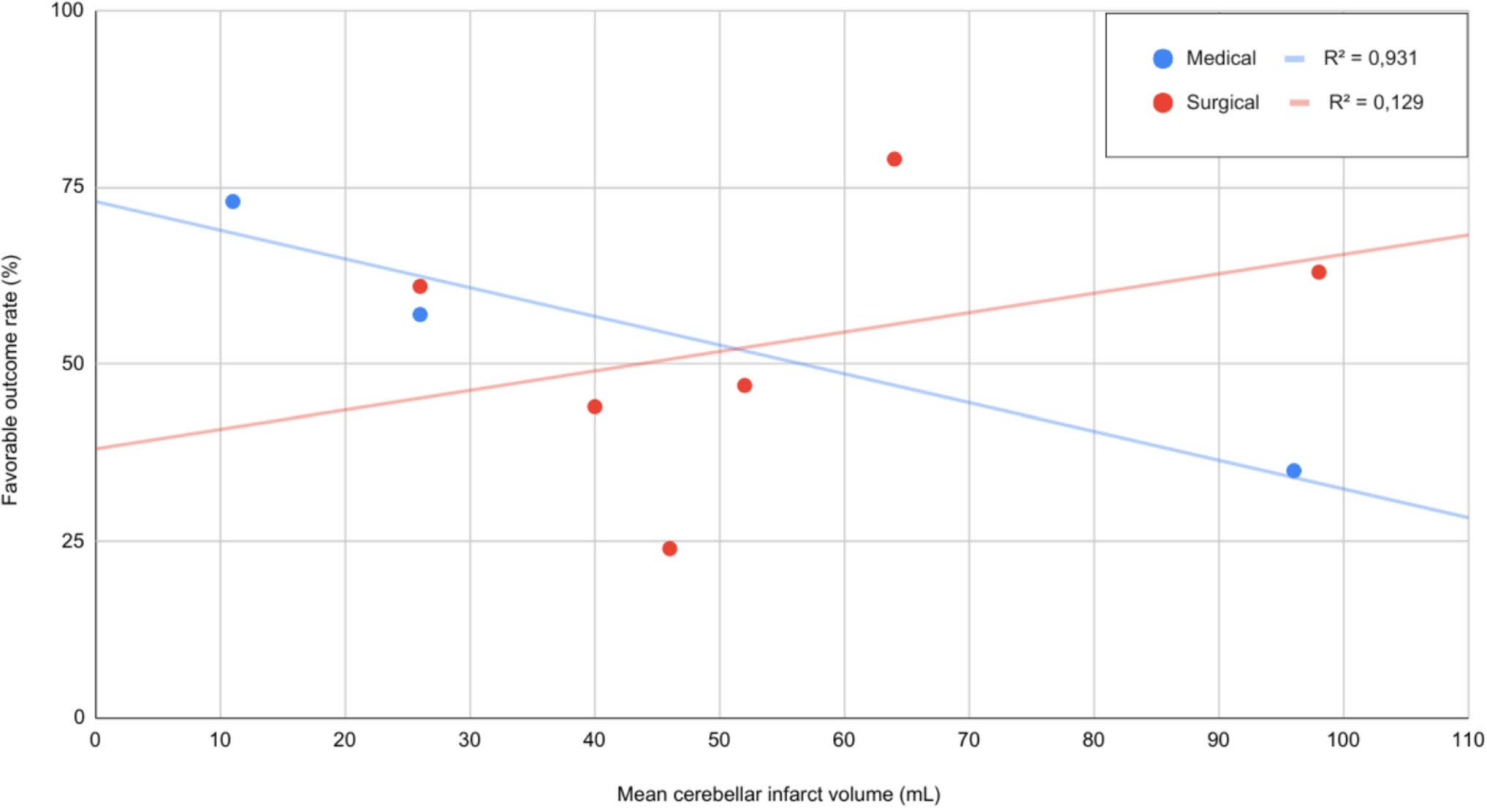
Cerebellar infarct volume and outcome. For patients with infarcts > 51 mL, surgical intervention (61.48%, 95% CI: 46.06–74.91) was associated with significantly higher rates of favorable outcome compared with medical therapy 35.00%, 95% CI: 20.63, 51.68; *p* = 0.018). Each dot represents an individual study cohort included in the analysis

In the medical group, larger infarct volume was significantly associated with worse outcomes (estimate = 1.1441, *p* < 0.0001). In the surgical group, infarct volume (estimate = −0.3570, *p* = 0.3867) was not significantly associated with outcomes Figure 2.

#### Glasgow coma scale score at presentation

**Fig. 3:**
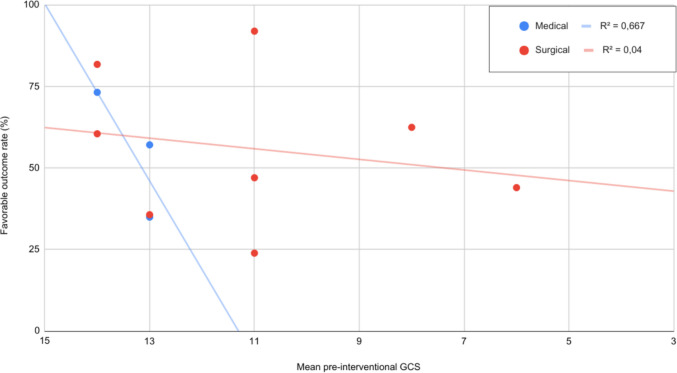
Presenting glassgow coma scale score and outcome. Patients with GCS ≤ 13 demonstrated improved outcomes following surgical intervention, relative to medical management alone (*p* < 0.05). Each dot represents an individual study cohort included in the analysis

In the surgical group, higher pre-interventional GCS showed a non-significant trend towards better outcomes (estimate = 0.0601, *p* = 0.5724) Figure 3.

#### Age at presentation

**Fig. 4:**
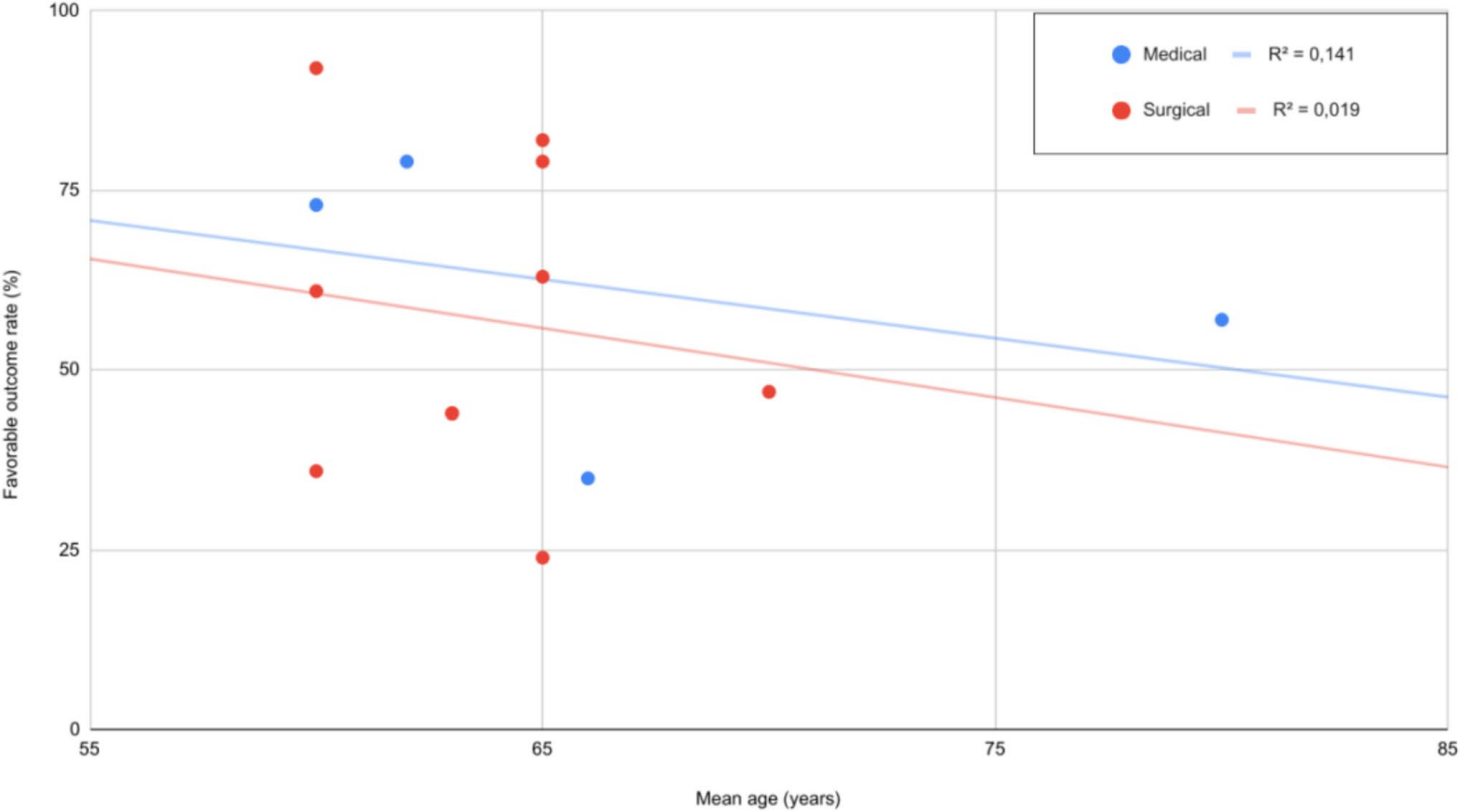
Age at presentation and outcome. Increasing age was associated with poorer outcomes across both treatment groups. As age increases, patients consistently experienced worse outcomes with surgical interventions than medical management alone (*p* < 0.05). Each dot represents an individual study cohort included in the analysis

Age was not significantly associated with outcomes in either the medical group (estimate = −0.0399, *p* = 0.3860) or the surgical group (estimate = −0.0284, *p* = 0.7495), although point estimates suggest a trend towards worse outcomes with increasing age Figure 4.

#### Time of symptom onset to surgical intervention

**Fig. 5:**
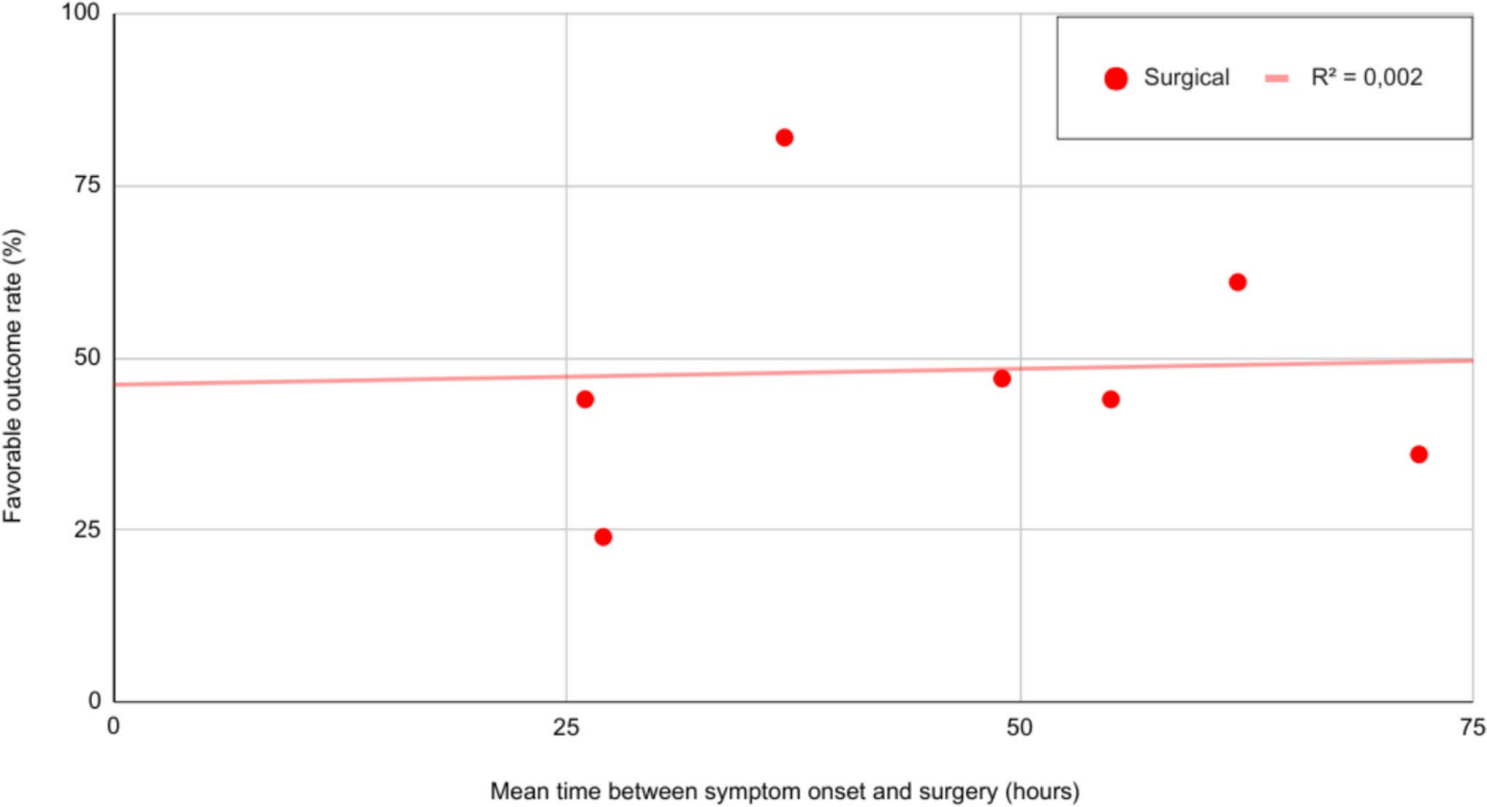
Time to surgery and outcome. Surgery performed within the first 72 h of symptom onset did not significant influence outcome (*p* = 0.93). Each dot represents an individual study cohort included in the analysis

Time from symptom onset to surgery in the surgical group had no significant impact on outcomes (estimate = −0.0021, *p* = 0.7911), indicating no measurable effect of surgical timing Figure 5.

#### Favorable outcomes and mortality rate by intervention type

Combination therapy with suboccipital decompressive craniectomy, necrosectomy, and external ventricular drainage (SDC-N-EVD) conferred the greatest benefit. Compared with medical therapy, SDC-N-EVD increased odds of a favorable outcome by 3.1-fold (95% CI: 1.18–8.14; *p* < 0.05). The only surgical intervention found to statistically reduce odds of favorable outcome, relative to medical management, was a stand-alone SDC (*p* < 0.05). SDC-N-EVD exhibited improved mortality rates against all other treatment types except medical management Tables 1 and 2.

Treatment-ranking analysis demonstrated that SDC-N-EVD had the highest probability of yielding a favorable outcome, followed by (2) medial management, (3) SDC-N, (4) EVD, (5) SDC-EVD, and (6) SCD. Regarding mortality, SDC-N-EVD ranked first (lowest mortality), with medial management ranking second; while EVD alone afforded the worst mortality rate. In summary, per rank probabilities of all management strategies for SOCI, SDC-N-EVD ranked first for providing the greatest likelihood of resulting in a favorable outcome and reduced mortality Table 3.

**Table 3:**
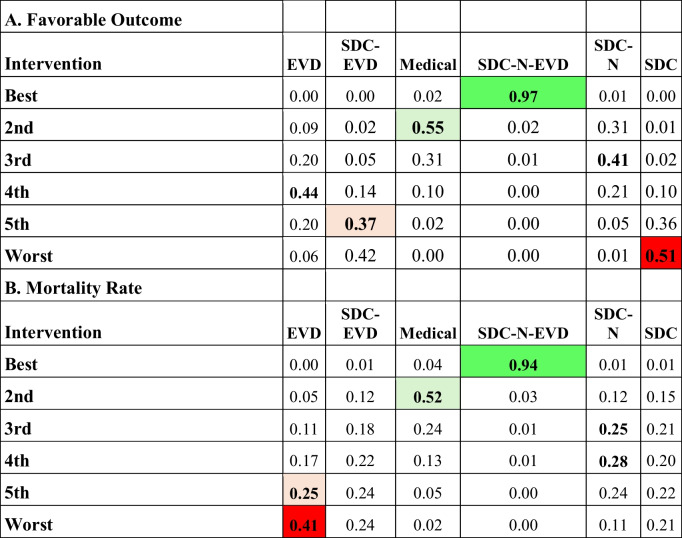
(A) Rank probability for favorable outcome. SDC-N-EVD exhibited the highest probability for a favorable outcome, followed by medical management. SDC alone, followed by SDC-EVD exhibited the two worst probabilities for favorable outcome. (B) Rank probability for mortality rate. SDC-N-EVD and medical management exhibited the highest probabilities for reduced mortality rate. EVD: external ventricular drain; N: necrosectomy; SDC: suboccipital decompressive craniectomy. Green shading indicates the intervention with the highest probability of having the most favorable outcome or reduced mortality rate (best rank), while red shading indicates the intervention with the greatest probability of being the worst outcome or greatest mortality rate (worst rank); light green (better rank) and red shading (worse rank) indicate high probabilities for intermediate probabilities

### Length of stay by intervention type

SDC-N-EVD was also associated with shorter length of stay compared with other treatment strategies, albeit statistical significance was not consistently met given suspected underpowering of analyses (Table 4). Rank-probability analysis however confirmed SDC-N-EVD as most likely to reduce hospitalization length of stay, followed by medical therapy at second Table 5.

**Table 4:**
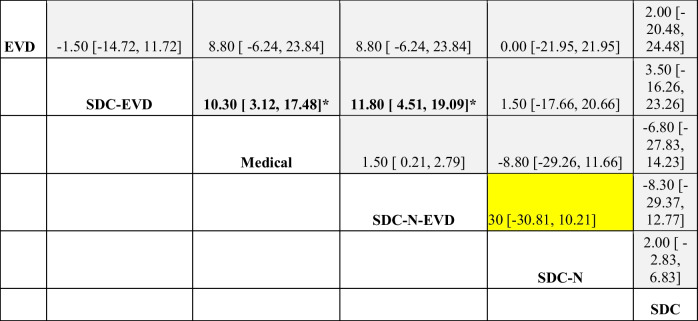
Length of stay odds per treatment cohort. Network meta-analysis for length of stay by indirect comparison of medical and surgical interventions. Overall, SDC-N-EVD exhibited improved odds of reducing length of stay compared to other treatment modalities, although statistical significance was not reached in each comparison. Odds ratio with 95% CI presented. *indicates statistical significance. EVD: external ventricular drain; N: necrosectomy; SDC: suboccipital decompressive craniectomy

**Table 5:**
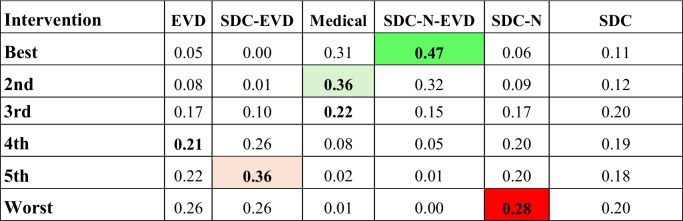
Rank probability for length of stay. SDC-N-EVD, followed by medical management yielded in the highest probability for reducing length of stay. Green shading indicates the intervention with the highest probability of being the most effective (best rank), while red shading indicates the intervention with the greatest probability of being the least effective (worst rank); light green (better rank) and red shading (worse rank) indicate high probabilities for intermediate probabilities of effectiveness. EVD: external ventricular drain; N: necrosectomy; SDC: suboccipital decompressive craniectomy

## Discussion

Provided the variability in management strategies for SOCIs, our investigation sought to provide clarity on interventional thresholds and determine whether an optimal treatment strategy exits. Overall, patients with larger infarction volumes derived greater benefit with surgical management—specifically, when managed with the combined approach of SDC-N-EVD. SDC-N-EVD was associated with the highest likelihood of favorable outcomes, lower mortality, and shorter hospitalization. In contrast, the timing of surgery from symptom onset did not significant alter outcomes.

### Medical versus surgical management

General comparison between medical and surgical cohorts exhibited no significant differences in outcome, yet when we stratified by intervention type, SDC-N-EVD exhibited a clear advantage over medical therapy. The improved outcomes from utilizing SDC-N-EVD is likely attributed to the combined effects of acutely addressing the hydrocephalus (i.e., lowering intracranial pressure), relieving posterior fossa mass effect, mitigating brainstem compression, and addressing altered cerebrospinal fluid dynamics postoperatively, while the necrosectomy may further attenuate the inflammatory sequelae of infarction [4, 7, 12, 22, 24].

On the contrary, standalone EVD was most likely to yield in mortality. The worse outcome is likely attributed to the risk of upward transtentorial herniation following ventricular decompression, which in turn can exacerbate brainstem compression [7, 12, 22, 24]. These findings underscore the importance of surgical comprehensiveness: interventions that address both mass effect and CSF circulation appear superior to temporizing measures alone.

### Infarction volume

Our results highlight infarct volume as a pivotal factor in treatment selection. Favorable outcomes with medical management declined linearly with increasing infarct size, whereas surgical outcomes remained relatively stable. Surgery conferred significant benefit over medical therapy.

Prior investigations have also recognized the critical nature of infarct volume on surgical decision making [10, 39]. For instance, some have suggested even at smaller infarct volumes (35 mL or greater) better outcomes are experienced with surgical decompression than an EVD alone [39]. Although we did not stratify outcomes by infarct volume for each specific surgical modality, our results reinforced the limited role of EVD alone given worse outcomes; one can surmise such a premise, asa suboccipital decompression with necrosectomy directly addresses the culprit (i.e., infarcted swollen cerebellar tissue yielding in mass effect), while an EVD alone simply temporizes the consequences (hydrocephalus) of the underlying problem [10, 39]. Nevertheless, there are considerable nuances in management where a standalone EVD can be considered, depending on patient exam, infarct volume, and radiographic data [10, 39].

### Pre-interventional GCS

Neurological status (i.e., clinical exam) remains a cornerstone of surgical decision making [17, 33]. Patients presenting with GCS ≤ 13 were more likely to benefit from surgery, whereas those with preserved consciousness (GCS 14–15) tended to fare better with medical management. Although direct comparisons with prior studies are limited, our results align with the American Heart Association/American Stroke Association guidelines recommending surgical intervention in patients with progressive neurological deterioration [37].

### Patient age

While age was inversely correlated with favorable outcome across all treatment modalities, outcomes between surgical versus medical therapy did not differ significantly within age strata. These findings suggest age alone should not preclude surgical intervention when otherwise indicated. Prior literature also supports such a conclusion, as there are reports of acceptable surgical outcomes in older SOCI patients, while parallel data from supratentorial infarctions demonstrates age does not independently dictate treatment benefit [33, 34, 37]. Hence, age can be used to inform discussions on treatment decision-making with patients and family, but alone age should not exclude a patient from an intervention if warranted.

### Time to surgery

Consistent with prior literature, our analysis did not identify difference in outcome between early and later surgical intervention—with outcomes equivalent if surgery was performed in the first 72 h [13, 16, 22, 23]. Although some have advocated for earlier decompression, current evidence—including American Heart Association/American Stroke Association guidelines—indicate the outcomes are primarily influenced by neurological status at the time of surgery, rather than precise timing, provided the intervention occurs prior to herniation [37].

### Length of stay

Despite being the most invasive strategy, SCD-N-EVD was associated with both improved outcomes and shorter hospitalization. By definitively addressing the cerebellar infarct, such allows for directly alleviating mass effect, reducing hydrocephalus, and preventing further neurologic deterioration, transitioning the disease course towards expedited clinical recovery; whereas conservatively managed patients require graduate stabilization and hence prolonged intensive care unit monitoring [15, 39].

### Limitations

There are several important limitations to note. The majority of included studies were retrospective in nature, with only one prospective cohort and a single randomized controlled trial, making the overall evidence base vulnerable to selection bias, incomplete data capture, and unmeasured confounding. Sample sizes were often small, and considerable heterogeneity existed across studies with respect to patient demographics, infarct volume thresholds, timing of intervention, surgical technique, and definitions of favorable outcome. The clinical and methodological diversity poses a challenge to the transitivity assumption required for network meta-analysis. Specifically, the indications for surgical intervention were not standardized; some centers operated prophylactically based on radiological criteria, while others adopted a *wait-and-watch* approach, reserving surgery for patients with clinical deterioration. Furthermore, variations in neurocritical care protocols—ranging from osmotherapy regimens to sedation strategies across different years and institutions—introduce a layer of clinical heterogeneity that may confound the direct and indirect comparisons between surgical modalities. While random-effects modeling was used to account for the above variability, there will nonetheless be residual heterogeneity in the final analysis.

Outcome reporting was inconsistent, with different studies employing various functional scales such as the Modified Rankin Scale or Glasgow Outcome Scale, and follow-up durations were frequently limited, thereby restricting the ability to assess long-term functional recovery, quality of life, or independence. Furthermore, the timing of surgery was not uniformly defined—some studies measured from symptom onset while others from hospital admission—and procedural details such as the extent of necrosectomy or the size of decompression were inconsistently reported, limiting comparability between surgical modalities. Restricting the analysis to English-language publications may also have introduced language bias, while publication bias remains possible despite funnel plot evaluation. Finally, most of the included studies were conducted in high-resource healthcare settings, which may limit generalizability to regions with fewer neurosurgical resources or different models of stroke care. These limitations highlight the need for larger, multicenter prospective studies and randomized controlled trials with standardized protocols to validate prognostic thresholds and refine treatment algorithms for space-occupying cerebellar infarctions.

## Conclusion

In conclusion, our systematic review and meta-analysis suggest surgical intervention is associated with improved outcomes for patients with SOCIs who presented with larger infarct volumes. Among the evaluated surgical strategies, combined SDC–N–EVD exhibited the highest likelihood of favorable outcomes and reduced hospitalization. Age and timing of surgery did not significantly modify outcomes, suggesting that these factors should not serve as absolute contraindications to intervention when clinically indicated—however, these findings must be interpreted in light of the significant heterogenetic amongst included studies. Nevertheless, these findings support the development of standardized, evidence-based treatment algorithms, and underscore the need for prospective multicenter studies to validate prognostic thresholds and optimize patient selection for surgical management of SOCI.

## Supplementary Information

Below is the link to the electronic supplementary material.

ESM 1 Supplementary Material 1 (DOCX 13.5 KB)

## Data Availability

No datasets were generated or analysed during the current study.

## Abbreviations

CI: Confidence interval
EVD: External ventricular drainage
GCS: Glasgow Coma Scale
OR: Odds ratio
SDC: Suboccipital decompressive craniectomy
SOCI: Space-occupying cerebellar infarction

## References

[1] Amar AP (2012) Controversies in the neurosurgical management of cerebellar hemorrhage and infarction. Neurosurg Focus FOC 32(4):E1 10.3171/2012.2.FOCUS11369 22463111

[2] Amarenco P et al (1994) Causes and mechanisms of territorial and nonterritorial cerebellar infarcts in 115 consecutive patients. Stroke 25(1):105–112 8266355 10.1161/01.str.25.1.105

[3] Bogousslavsky J, Van Melle G, Regli F (1988) The Lausanne Stroke Registry: analysis of 1,000 consecutive patients with first stroke. Stroke 19(9):1083–1092 3413804 10.1161/01.str.19.9.1083

[4] Hernandez-Duran S et al (2024) Necrosectomy versus stand-alone suboccipital decompressive craniectomy for the management of space-occupying cerebellar infarctions-a retrospective multicenter study. Neurosurgery 94(3):559–566 37800900 10.1227/neu.0000000000002707

[5] Higgins JP et al (2011) The Cochrane Collaboration’s tool for assessing risk of bias in randomised trials. BMJ. 10.1136/bmj.d5928 10.1136/bmj.d5928 PMC3196245 22008217

[6] Hornig CR et al (1994) Space-occupying cerebellar infarction. Clinical course and prognosis. Stroke 25(2):372–374 8303748 10.1161/01.str.25.2.372

[7] Ito M et al (1994) Surgical management of comatose patients with cerebellar infarction. J Clin Neurosci 1(4):251–256 18638769 10.1016/0967-5868(94)90065-5

[8] Jauss M et al (1999) Surgical and medical management of patients with massive cerebellar infarctions: results of the German-Austrian Cerebellar Infarction Study. J Neurol 246(4):257–264 10367693 10.1007/s004150050344

[9] Jüttler E et al (2009) Long-term outcome after surgical treatment for space-occupying cerebellar infarction: experience in 56 patients. Stroke 40(9):3060–3066 19574554 10.1161/STROKEAHA.109.550913

[10] Kapapa T et al (2024) Volumetry as a criterion for suboccipital craniectomy after cerebellar infarction. J Clin Med. 10.3390/jcm13195689 39407749 10.3390/jcm13195689 PMC11477441

[11] Kase CS et al (1993) Cerebellar infarction. Clinical and anatomic observations in 66 cases. Stroke 24(1):76–83 8418555 10.1161/01.str.24.1.76

[12] Kayan Y et al (2019) Current endovascular strategies for posterior circulation large vessel occlusion stroke: report of the Society of NeuroInterventional Surgery Standards and Guidelines Committee. J Neurointerv Surg 11(10):1055–1062 31103994 10.1136/neurintsurg-2019-014873

[13] Kim MJ et al (2016) Preventive Suboccipital Decompressive Craniectomy for Cerebellar Infarction: A Retrospective-Matched Case-Control Study. Stroke 47(10):2565–2573 27608818 10.1161/STROKEAHA.116.014078

[14] Kudo H et al (2007) Controversy of surgical treatment for severe cerebellar infarction. J Stroke Cerebrovasc Dis 16(6):259–262 18035243 10.1016/j.jstrokecerebrovasdis.2007.09.001

[15] Kumral, Emre and Dorukoğlu, Mesut and Orman, Mehmet and Özgiray, Erkin, Decompressive Craniectomy in Patients with Malignant Cerebellar Infarction a Randomized, Controlled Trial (Demci Trial). Available at SSRN: https://ssrn.com/abstract=4423441, 10.2139/ssrn.4423441

[16] Lindeskog D et al (2019) Long-term functional outcome after decompressive suboccipital craniectomy for space-occupying cerebellar infarction. Clin Neurol Neurosurg 176:47–52 30522035 10.1016/j.clineuro.2018.11.023

[17] Lucia K et al (2023) Predictors of clinical outcomes in space-occupying cerebellar infarction undergoing suboccipital decompressive craniectomy. Front Neurol 14:1165258 37139059 10.3389/fneur.2023.1165258 PMC10149688

[18] Macdonell RA, Kalnins RM, Donnan GA (1987) Cerebellar infarction: natural history, prognosis, and pathology. Stroke 18(5):849–855 3629642 10.1161/01.str.18.5.849

[19] Mathew P et al (1995) Neurosurgical management of cerebellar haematoma and infarct. J Neurol Neurosurg Psychiatry 59(3):287–292 7673958 10.1136/jnnp.59.3.287 PMC486032

[20] Moher D, Liberati A, Tetzlaff J, Altman DG (2009) PRISMA Group. Preferred reporting items for systematic reviews and meta-analyses: the PRISMA statement. PLoS Med. 6(7):e1000097. 10.1371/journal.pmed.1000097 10.1371/journal.pmed.1000097 PMC2707599 19621072

[21] Mostofi K (2013) Neurosurgical management of massive cerebellar infarct outcome in 53 patients. Surg Neurol Int 4:28 23532804 10.4103/2152-7806.107906 PMC3604818

[22] Neugebauer H et al (2013) Space-occupying cerebellar infarction: complications, treatment, and outcome. Neurosurgical Focus FOC 34(5):E8 10.3171/2013.2.FOCUS12363 23634927

[23] Pfefferkorn T et al (2009) Long-term outcome after suboccipital decompressive craniectomy for malignant cerebellar infarction. Stroke 40(9):3045–3050 19574555 10.1161/STROKEAHA.109.550871

[24] Raco A, Caroli E, Isidori A, Salvati M (2003) Management of acute cerebellar infarction: one institution's experience. Neurosurgery, 53(5), 1061–5. 10.1227/01.neu.0000088766.34559.3e 10.1227/01.neu.0000088766.34559.3e 14580272

[25] Salanti G, Ades AE, Ioannidis JP (2011) Graphical methods and numerical summaries for presenting results from multiple-treatment meta-analysis: an overview and tutorial. J Clin Epidemiol 64(2):163–171 20688472 10.1016/j.jclinepi.2010.03.016

[26] Shamseer L et al (2015) Preferred reporting items for systematic review and meta-analysis protocols (PRISMA-P) 2015: elaboration and explanation. BMJ. 10.1136/bmj.g7647 25555855 10.1136/bmj.g7647

[27] Sterne JA et al (2016) ROBINS-I: a tool for assessing risk of bias in non-randomised studies of interventions. BMJ 355:i4919 27733354 10.1136/bmj.i4919 PMC5062054

[28] Sterne JAC et al (2019) RoB 2: a revised tool for assessing risk of bias in randomised trials. BMJ 366:l4898 31462531 10.1136/bmj.l4898

[29] Suyama Y et al (2019) Evaluation of clinical significance of decompressive suboccipital craniectomy on the prognosis of cerebellar infarction. Fujita Med J 5(1):21–24 35111496 10.20407/fmj.2018-010 PMC8766232

[30] Tartara F et al (2018) Strokectomy and extensive cerebrospinal fluid drainage for the treatment of space-occupying cerebellar ischemic stroke. World Neurosurg 115:e80–e84 29625312 10.1016/j.wneu.2018.03.178

[31] Taylor DR et al (2020) Predicting Surgical Intervention in Cerebellar Stroke: A Quantitative Retrospective Analysis. World Neurosurg 142:e160–e172 32599209 10.1016/j.wneu.2020.06.156

[32] Tohgi H et al (1993) Cerebellar infarction. Clinical and neuroimaging analysis in 293 patients. The Tohoku cerebellar infarction study group. Stroke 24(11):1697–1701 8236346 10.1161/01.str.24.11.1697

[33] Tsitsopoulos PP et al (2011) Surgical treatment of patients with unilateral cerebellar infarcts: clinical outcome and prognostic factors. Acta Neurochir (Wien) 153(10):2075–2083 21833781 10.1007/s00701-011-1120-4

[34] van der Worp HB et al (2021) European Stroke Organisation (ESO) guidelines on the management of space-occupying brain infarction. Eur Stroke J 6(2):Xc–cx 34414308 10.1177/23969873211014112 PMC8370072

[35] Villalobos-Díaz R et al (2022) Characteristics and long-term outcome of cerebellar strokes in a single health care facility in Mexico. Cureus 14(9):e28993 36259000 10.7759/cureus.28993 PMC9573303

[36] Wang Y et al (2022) Rate of infarct-edema growth on CT predicts need for surgical intervention and clinical outcome in patients with cerebellar infarction. Neurocrit Care 36(3):1011–1021 34966956 10.1007/s12028-021-01414-x PMC9110544

[37] Wijdicks EF et al (2014) Recommendations for the management of cerebral and cerebellar infarction with swelling: a statement for healthcare professionals from the American Heart Association/American Stroke Association. Stroke 45(4):1222–1238 24481970 10.1161/01.str.0000441965.15164.d6

[38] Winslow N et al (2023) Posterior fossa ischemic infarction: single-center retrospective review of non-surgical and surgical cases. Neurosurg Rev 46(1):35 36629928 10.1007/s10143-022-01939-5

[39] Won SY et al (2024) Functional outcomes in conservatively vs surgically treated cerebellar infarcts. JAMA Neurol 81(4):384–393 38407889 10.1001/jamaneurol.2023.5773 PMC10897822

