# Supplementary material for: Comparison of neurosurgical and medical management options of space-occupying cerebellar infarction
Source: Acta Neurochir (Wien). 2026 Mar 6;168(1):60. doi: 10.1007/s00701-026-06809-3 (PMC12967541; doi:10.1007/s00701-026-06809-3)
Supplement: Supplementary file 1 — Supplementary Material 1 (DOCX 13.5 KB) [file 701_2026_6809_MOESM1_ESM.docx]

**Supplementary Methods S1:** Search strategy

(cerebellum OR cerebellum OR cerebellar) AND (infarctation OR infarcted OR infarctic OR infarcting OR infarction OR infarction OR infarct OR infarctions OR infarcts OR infarctive) AND (craniotomy OR craniotomy OR craniotomies OR (craniotomy OR craniotomy OR craniectomies OR craniectomy) OR (necrosectomies OR necrosectomy) OR strokectomy OR (craniotomy OR craniotomy OR craniectomies OR craniectomy) OR (craniotomy OR craniotomy OR craniotomies) OR (decompress OR decompressed OR decompresses OR decompressing OR decompression OR decompression OR decompressions OR decompressive))
